# Examining the impact of physical education and physical skills development on preschoolers’ physical and mental health

**DOI:** 10.3389/fpsyg.2022.1000653

**Published:** 2023-01-06

**Authors:** Nina Wang, Qinglei Wang, Xiaohong Liu, Masood Mahfooz, Zubia Savila

**Affiliations:** ^1^Faculty of Education, Department of Educational Psychology and Counseling, University of Malaya, Kuala Lumpur, Malaysia; ^2^Faculty of Sports and Exercise Science, University of Malaya, Kuala Lumpur, Malaysia; ^3^Faculty of Education, Henan Normal University, Xinxiang, Henan, China

**Keywords:** preschooler, physical and mental health, physical skills development, physical education, comprehensive teaching

## Abstract

With greater socio-economic development and the popularization of scientific child-rearing methods, education has become of primary importance in contemporary society. This study attempts to promote the physical and mental health of preschoolers and improve their comprehensive learning ability. To understand the impact of sports skills and Physical Education (PE) on preschoolers’ physical and mental health, we utilized the Questionnaire Survey (QS) and expert evaluation. First, the development of sports skills and the basic connotation of sports were expounded. Second, the characteristics of preschoolers and the importance of preschool education were discussed. Finally, the physical and mental health of 60 preschoolers was evaluated based on physical skill development and sports. The results revealed that the respondent preschoolers were grouped reasonably, and the research results had high reference values. In the control group, the physical conditions of four preschoolers changed significantly in the best case. By comparison, in the experimental group, preschoolers with significant physical changes had reached nine at best. In addition, as high as nine respondents showed obvious improvement in their mental state in the best case. Therefore, this study demonstrates that physical skill development and PE significantly impact preschoolers’ physical and mental health, which has an important impact on preschoolers’ learning. This finding provides a reference for preschoolers’ sports skills development and contributes to their comprehensive PE teaching.

## 1. Introduction

Sports involves physical exertion and skills, feature competitiveness, and cultivate competitive consciousness. Students can enjoy competition by participating in or organizing sports activities (Garibaldi and Russell, 2021). As the main content of basic education, preschool education plays an important role in the overall development of children. For example, preschool education can strengthen physical skills teaching (Pérez-Ordás et al., 2021). Thus, it is essential in promoting overall social education, and effective preschool education can stimulate students’ interest in learning and lay a solid foundation for their overall growth. Unsurprisingly, the research on preschool education has aroused worldwide attention.

Narzikulovich (2022) pointed out that sports activities were often operational and diverse, so students could explore various ways of playing. In the process of exploration, students would feel the joy of success and their self-awareness and self-confidence would also increase. Tashpulatov and Shermatov (2021) held that preschoolers were the motherland’s future, and that the body was the capital of future life, study, and work. Physical Education (PE) is inseparable from preschoolers’ lives, and so it is crucial to ascertain the educational form of kindergarten sports activities (Tashpulatov and Shermatov, 2021). Pasichnyk et al. (2021) suggested that art, physical, and music education had increasingly received attention in the context of comprehensive quality education. Among them, PE was directly related to developing students’ physical and mental quality and occupied an essential position in students’ growth. With the development of comprehensive quality education, more attention has been paid to PE. Although it is supposed to be fun and stimulating, PE has become a burden in the eyes of students under traditional PE teaching methods. As a new model of PE, the concept of “happy sports” promotes students’ interest in sports and sports and improves their personalities. By integrating “happy sports” into PE in preschool, the teaching quality can be significantly improved (Pasichnyk et al., 2021).

This study first examines the development of sport skills and examines the basic connotations of sports. Subsequently, the characteristics of preschoolers and the importance of preschool education are discussed. Finally, the comprehensive impact of physical education on the minds and bodies of preschoolers is investigated. The innovation of this study lies in the breakthrough research on the group of preschoolers and the innovative exploration of the subject by using two methods of expert assessment and questionnaire survey. The findings provide a reference to enhance the physical education of preschoolers and contributes to their comprehensive educational progress.

## 2. Research theory

### 2.1. Physical skills development and PE

With the increased emphasis on PE, it has become vital in developing students’ comprehensive quality (Tan et al., 2021). PE has become an essential course in school education under the requirements of quality education and its importance is mainly manifested in the following aspects. First, it is a necessary guarantee to improve students’ physical quality. PE is suitable for exercising students’ bodies, and allowing them to rest their brains and eyes from heavy schoolwork. Physical exercise creates a muscular healthy physique. Second, PE is an effective way to sharpen the spiritual will of students. It enables them to overcome challenges through practice and to learn healthy competition. Third, PE improves the moral quality of students. In PE courses, many excellent moral qualities such as solidarity and mutual assistance are subtly absorbed by students through practice (Gafurova and Ruzimbaev, 2021).

Physical skills refer to the ability of students to complete physical movements in PE courses and during physical exercise. Such skills reflect the effect of PE and health teaching courses, mainly in terms of the physical abilities of students. This aspect is essential because physical and motor skills are significant factors in accomplishing other learning objectives (Ruzimbaevich and Ruzimbaev, 2021). Besides, physical skills are also the main content of the PE courses (Plummer et al., 2021). Mannino et al. (2019) investigates the causal and direct relationship between sport activity and performance.

There are many classifications of sports skills, usually divided into six categories. The first is the speed-strength type such as sprinting, weightlifting, speed skating, and throwing. The second is endurance in the form of race walking, long-distance running, and swimming. The third is the phenotype, such as gymnastics, figure skating, and synchronized swimming. The fourth is precision skills such as shooting and archery. The fifth is the net confrontation type: tennis, table tennis, and badminton. The sixth is the same-court confrontation type such as basketball, rugby, and ice hockey. These categories of physical skills are based on the type of physical function of the learner, and educators need to clearly understand it to teach effectively (Sevimli-Celik, 2021).

### 2.2. Preschoolers

The term preschoolers refers to children who have not yet reached school age. From a global perspective, countries have different regulations regarding the age of children entering school, which is generally 6 or 5 years old. Thus, the age limit of preschoolers can be different (Berdiyev, 2021). Chinese children go to school at the age of six. In a certain period, the statistical information on preschoolers is necessary for the country to develop childcare and arrange healthcare institutions (Navarro-Patón et al., 2021). The study of preschoolers must first take into account their developmental characteristics as it would be conducive to their education. For example, learning materials and methods must be selected according to children’s cognitive aspects (Fernández-Valero et al., 2021). Parents and kindergarten teachers can guide children in social learning and practice with understanding, patience, and firmness according to preschoolers’ emotional characteristics. In short, changes in preschoolers are rapid and regular, and once this fact is recognized, appropriate methods can be used to prepare children accordingly (Dobell et al., 2021).

A common characteristic of preschoolers is that they are active and playful. Although children play in various ways in different cultures, they emulate the activities of adults around them. These activities provide children with many opportunities to interact with people and objects (Beknazarovich, 2022). Children’s social factors outside the family are formed at the age of two, and during this period they prefer to be alone and enjoy themselves. They lack the awareness of communicating with others (Riyanto and Syamsudin, 2021). At this age, children always preempt what they want (Mischenko et al., 2021). They begin to make friends at the age of three and acquire basic social skills. They like to please adults and respond positively to appropriate guidance on social behavior from adults. At the age of four, children enjoy playing with their peers, being creative, and interacting with each other (Demchenko et al., 2021).

To sum up, the teaching content for preschoolers must start from practice. Preschool teaching is the main period in which students’ learning characteristics are formed. Furthermore, physical skills are also the main content of current social teaching. Therefore, training preschoolers in physical skills is a very reasonable and promising decision (Terrón-Pérez et al., 2021). However, the implementation of this decision needs to be considered in the teaching process. Additionally, it is imperative to focus on the impact of physical skills teaching on preschoolers’ physical and mental health (Rakhmatillayevna, 2021).

## 3. Research methods

### 3.1. Research necessity outlook

**FIGURE 1:**
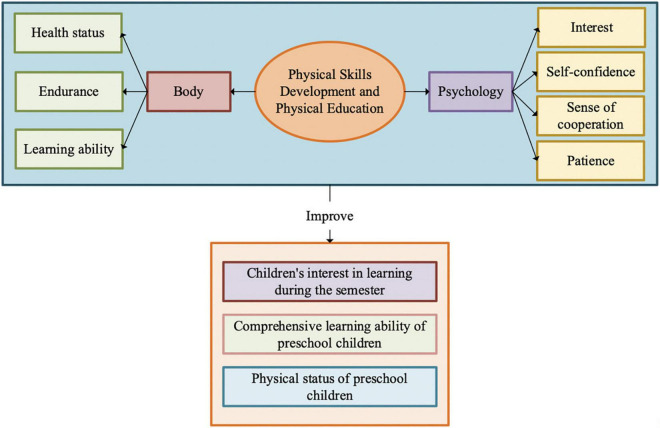
The impact of physical skills and PE on preschooler.

This study investigates the development of preschoolers’ sports skills and the impact of PE on their physical and mental health (Mecías-Calvo et al., 2021). Physically, sports skills teaching affects preschoolers’ sports competency, including endurance and physical strength. Meanwhile, the psychological impact of sports skill teaching on preschoolers is also very positive (Cordovil et al., 2021). Figure 1 illustrates the influence of sports skills development and PE on preschoolers.

Figure 1 indicates that it is essential to deeply research the physical and mental impact of physical on preschoolers. Thereby, this study mainly examines the influence of physical skills and education on the physical and mental health of current preschoolers using the literature and questionnaire survey.

### 3.2. Literature survey

The research participants are preschoolers, so the relevant literature is related to them. Physical skills and education are secondary factors. The literature search and analysis are carried out on these two aspects. After the literature review, the specific research direction and content can be determined, and the necessity of the current physical skills, PE, and preschool education can be analyzed. Furthermore, the influence of current physical skills and PE on preschoolers’ physical and mental health must be ascertained to reflect the role of physical skills and PE in preschool education.

### 3.3. Questionnaire survey

The questionnaire survey method is the main method of this study. Before the comparative analysis, the verification indicators should be determined. Therefore, based on consulting substantial relevant materials are combined with the development of sports physiology, sports measurement, and evaluation, and other disciplines to analyze sports skills and PE. First, the evaluation indicators are determined accordingly to ensure the scientific rationalization of experimental evaluation. The main objects of this questionnaire are preschool education experts. The experts are all from the education bureau of a certain city. The questionnaire investigates the effect of physical skills and PE on preschoolers’ physical and mental health. A total of 15 experts were surveyed, and 15 valid questionnaires were recovered. A questionnaire was also given to parents of students in a kindergarten class. Through parents, preschoolers’ physical, and mental changes in physical skills and PE can be directly understood. Sixty questionnaires were distributed to parents and 60 valid questionnaires were recovered. In short, this study conducted a survey and evaluation of 60 preschoolers to explore the impact of physical skills development and PE on their physical and mental health. Table 1 presents the results of the reliability and validity analysis of the questionnaire survey.

**TABLE 1:** The results of the reliability and validity analysis of the questionnaire survey.

Questionnaire	Very reasonable	Reasonable	Unreasonable
Content	12	3	0
Structure	14	1	0
Object	15	0	0
Overall	14	1	

In Table 1, the reliability and validity test is basically qualified, so the design of this questionnaire’s research method is reasonable. Preschoolers who were taught six physical skills were surveyed. The main physical and mental health effects of current physical skills teaching on preschoolers were determined by analyzing the teaching of different physical skills. The preschoolers in the first group was the control group and did not receive physical skills teaching during the investigation period. The physical skills teaching items of the other five groups of preschoolers’ were basketball, badminton, archery, swimming, and running. The preschoolers’ teaching changes during 20 days were investigated and evaluated. This study mainly uses Statistical Product and Service Solutions (SPSS) software and Excel to carry out a statistical analysis of the collected information. The impact of current physical skills and PE on preschoolers’ physical and mental health has been comprehensively studied through investigation and analysis. The result provides a reference in improving the current physical skills and the effect of PE.

## 4. Research results

### 4.1. Assessment of the condition of physical skills in preschoolers

**FIGURE 2:**
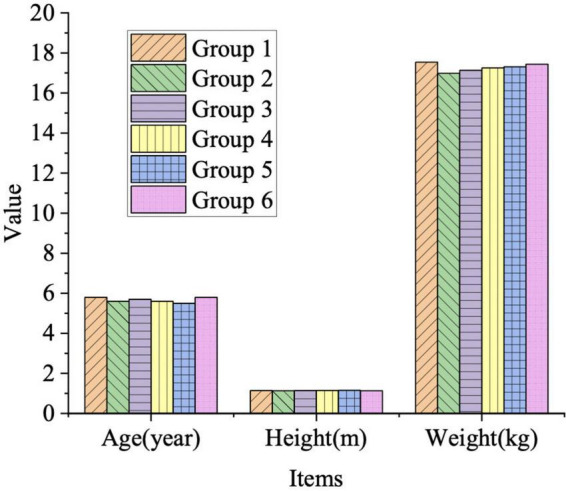
Average results of basic physical information of preschoolers.

The rationale for the research subjects is first determined before the study, and the subjects are grouped. The information for preschoolers is determined through a survey of their parents. The preschoolers are randomly divided into groups, and the teaching effect is examined by teaching different physical skills as the goal. Figure 2 and Table 2 present the basic physical information assessment of preschoolers.

**TABLE 2:** Evaluation results of basic information scope of preschooler.

Group	Age (year)	Height (m)	Weight (kg)
1	5.83 ± 0.53	1.14 ± 0.03	17.54 ± 1.82
2	5.62 ± 0.23	1.13 ± 0.06	16.98 ± 2.14
3	5.71 ± 0.41	1.14 ± 0.04	17.14 ± 1.95
4	5.67 ± 0.46	1.15 ± 0.04	17.25 ± 1.74
5	5.59 ± 0.57	1.16 ± 0.03	17.31 ± 1.85
6	5.81 ± 0.49	1.13 ± 0.05	17.44 ± 1.87
*P*	0.895	0.806	0.952

In Figure 2 and Table 2, the *P*-values are all greater than 0.05 in the evaluation results of the significance of basic physical information of preschoolers. There is little difference in basic physical information between the groups. Therefore, the grouping of students in the survey is very reasonable, which reduces the error of the survey and improves the practical value of the survey results.

### 4.2. The influence of physical skills of preschoolers on physical health

**FIGURE 3:**
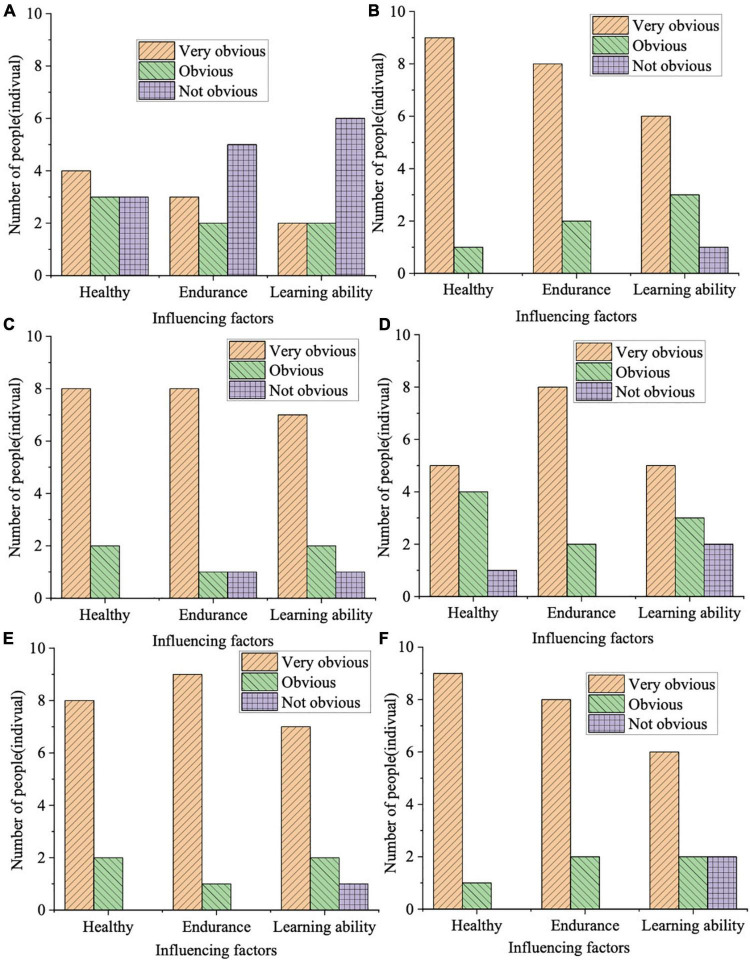
Assessment of the impact of physical skills teaching on the physical aspects of preschoolers [Panels **(A–F)** indicate the control group, basketball, badminton, archery, swimming, and running groups, respectively].

The main task of this study is to assess the physical and mental changes in preschoolers. The most obvious impact of physical skills on preschoolers is physical. Among the five physical skills, basketball, badminton, archery, swimming, and running have diverse effects. Thus, physical changes must be assessed through divergent aspects such as health, endurance, and learning ability. Figure 3 reveals the results of the impact of teaching physical skills on the physical abilities of preschoolers.

In Figure 3, on assessing the impact of teaching physical skills on the physical aspects of preschoolers, the evaluation results of the control group are not ideal. Only four preschoolers without receiving physical skills teaching have shown significant changes in physical conditions, and three respondents manifest no significant change at all. The number of students with a very significant change in endurance is 3, and the number of students with significant and insignificant changes is 2 and 5, respectively. The number of students with a very significant change in learning ability is 2, and that of students with significant and insignificant changes is 2 and 6, respectively. In the experimental groups, preschoolers who receive physical skills training present significant changes in endurance, health, and learning ability. Specifically, the number of students with a very significant overall change in physical status is at most 9 and at lowest 5. The number of students with insignificant changes is at most 2 and at lowest 0. Thus, teaching physical skills has a great influence on the physical aspects of preschoolers.

### 4.3. The influence of physical skills of preschoolers on mental aspects

**FIGURE 4:**
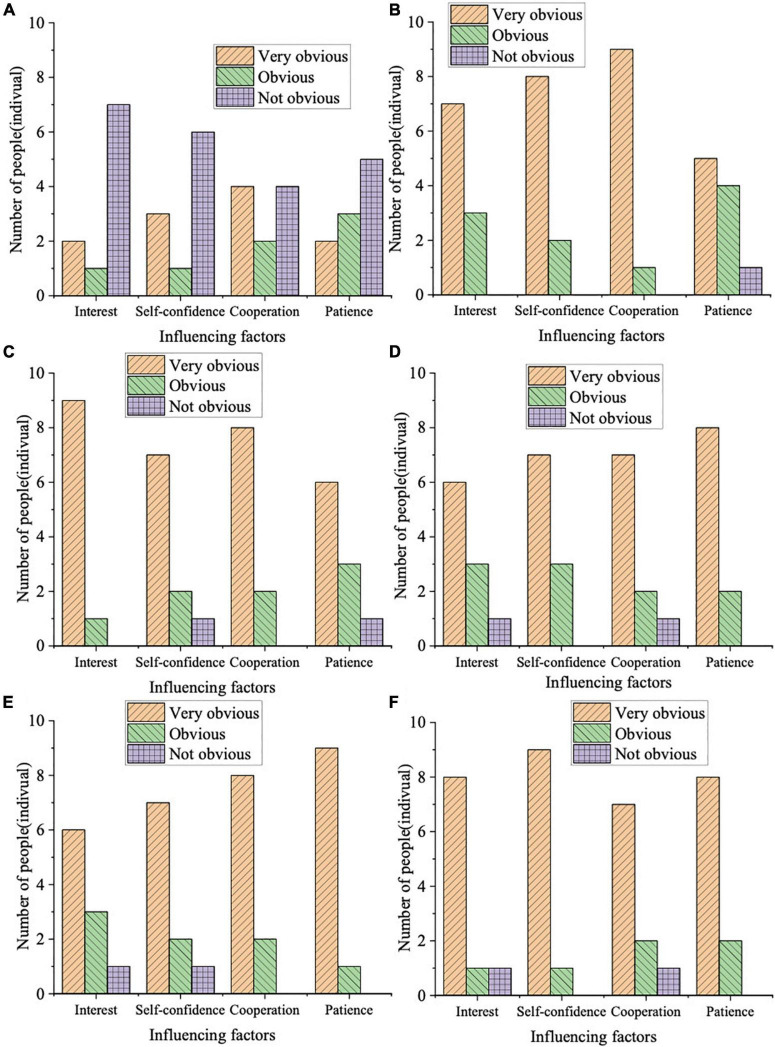
Assessment of the impact of physical skills teaching on the mental aspects of preschoolers [Panels **(A–F)** are the control group, basketball, badminton, archery, swimming, and running groups, respectively].

Psychological characteristics should be paid attention to in the mental care of preschoolers. The literature reviewed suggests a significant, and sometimes substantial, relationship between physical activity and later cognitive function and dementia (Kramer et al., 2006). At this age, children become capable of abstract thinking, and they can understand abstract concepts. Their creative imagination has also appeared; they can draw novel pictures and make up interesting stories. Thus, the teaching process should create a good atmosphere for Preschoolers. Educators enable preschoolers to feel truth, goodness, and beauty in their daily lives and the good qualities of the children can be cultivated in school. Children’s psychology develops positively in a healthy, positive, and loving environment. In teaching physical skills, this study ascertains the influence on the psychology of preschoolers. Specifically, it includes interest, self-confidence, cooperation awareness, and patience in preschoolers. Figure 4 displays the assessment results of the impact of teaching physical skills on the mental aspects of preschoolers.

Figure 4 demonstrates that in evaluating the influence of physical skills teaching on preschooler’s psychology, the evaluation results of the control group are not ideal. Two preschoolers without reaching physical skills teaching have shown very significant change in learning interest. Meanwhile, 1 and 7 respondents demonstrate a significant and insignificant change, respectively. In terms of self-confidence, the number of students who have shown a very significant, significant, and insignificant change is 3, 1, and 6, respectively. In cooperation awareness, the number of students with a very significant, significant, and insignificant change is 4, 2, and 4, respectively. Regarding patience, the number of students who have very significant, significant, and insignificant change is 2, 3, and 5, respectively. By comparison, the experimental group that received physical skills training showed obvious changes in interest, self-confidence, cooperation awareness, and patience. The number of students with a very significant overall change in mental state is at most 9 and at lowest 6, and the number of students with insignificant changes is at most 1 and at lowest 0. This result illustrates that learning physical skills has a great influence on the physical health of preschoolers. Compared with the study of Yan et al. (2022), this study provides a more comprehensive research perspective and content. Simultaneously, more methods are used, and the expert assessment is more scientific and reasonable for the research results (Yan et al., 2022).

## 5. Discussion

Preschool education is a critical period for the physical and mental development of children. Physical movement plays an important role in the cognitive and emotional development, and the personality formation of students in preschools. Additionally, their mental state is also related to the development of sports skills. The most obvious and direct impact of sports skills development on the perception of preschools is devolution (Senol, 2021). Decentralization, as opposed to self-awareness, helps to eliminate the psychological awareness that children tend to act on their self-knowledge and their own bodies. The preschool period, especially early childhood, is a key stage of decentralization, and the development of sports skills plays a unique role during this period (Young et al., 2021). In addition to helping children learn about themselves, a successful physical activity must also help them familiarize themselves with their environment and distinguish themselves from objects. Therefore, they can think and participate in physical activities according to the nature and characteristics of objects (Martínez-Bello and Estevan, 2021). A typical example is a game of hide and seek. Some children may only hide their heads for the first few rounds, thinking they will not be seen. After several rounds, they gradually learn to integrate themselves into the environment and are able to consider the game from their own perspective and context (Turdimurodov, 2021).

Moreover, sports emotion is a vital psychological factor affecting sports teaching in preschools. All emotions and emotional experiences associated with sporting activities in preschools can be called sporting emotions. Sports emotion can be subdivided into motor emotion, sports passion, and sports enthusiasm. Motor emotion is a persistent emotional state that involves the entire movement process (Honrubia-Montesinos et al., 2021). On the one hand, it can be assumed that a child’s basic sporting qualities are good and in this case, he or she is likely to be in good motor emotion, positively predisposed toward sports, and happy when participating in sports (Chang and Lei, 2021). Motor emotion, on the other hand, motor emotion is determined by basic physical quality, a period of physical condition, and some experience of success, happiness, failure, or sadness. Sports passion is a strong and rapid emotional experience (Yoshimi et al., 2021). An unexpectedly strong stimulus will trigger sports passion. For example, when a child with poor ability gets unexpected success in sports or when they are praised by teachers and classmates, it will trigger the child’s ecstasy and excitement. Finally, sports enthusiasm is a positive, powerful, stable, and profound emotional experience of sports (Bano et al., 2021).

Accordingly, this study investigates the influence of preschools’ sports skill development and PE on physical and mental health. The results demonstrated that when evaluating the influence of PE on preschools’ psychology, the evaluation result of the control group was not ideal. The two students who did not attend the PE course showed significant changes in learning interest. In contrast, the experimental group, which was taught physical skills, presented obvious changes in interest, confidence, cooperation, and patience. The maximum number of students with significant changes in their mental state was 9, and the maximum number of students with no obvious variations was 1, thus indicating that the teaching of physical skills had a great impact on the physical health of preschool children.

Therefore, this study demonstrates that preschools can play a crucial role in developing the physical skills of students and can provide certain guarantees for improving their physical health. This finding provides support for the healthy growth of preschool students and contributes to the improvement of preschool education.

## 6. Conclusion

Based on the continuous popularization of education levels, preschool education has become the main social education branch. However, the current preschool education is not perfect in sports skills and PE, so more research is needed to provide a reference. First, this study discusses the teaching connotation of sports skills. Subsequently, the basis and characteristics of the preschooler division are expounded. Finally, an investigation is conducted based on the influence of sports skills development and PE on preschoolers’ physical and mental health. The results testify that physical skill teaching significantly impacts students’ the physical state of students. First, in the control group, two preschoolers’ physical states changed obviously in the worst scenario, with six preschoolers without significant improvement. At the same time, four preschoolers’ physical state has shown significant improvement in the best case, with three showing no significant changes. Second, under the worst scenario, five preschoolers have significantly improved their physical state in the experimental group, with two preschoolers showing no significant changes. In the best scenario, these numbers are nine and zero, respectively. Further, the influence of sports skill teaching on preschoolers’ psychology is verified. Consequently, in the control group, two respondents’ mental states have remarkably changed, with seven showing no significant changes in the worst scenario. In the best case, four respondents’ mental state has shown significant improvement, with four presenting no significant improvement. In the control group, these numbers are six and one in the worst scenario and nine and zero in the best case. Therefore, sports skills teaching has a great impact on preschoolers’ physical and mental aspects and also on their learning. By strengthening preschoolers’ sports skill teaching, and improving their physical and psychological health, it will provide important support for their future comprehensive learning ability. Although this study provides relatively perfect research results, the design of research factors is not perfect. Further investigations are necessary to strengthen the research on preschoolers’ sports skills teaching and ascertain how it can improve the comprehensive teaching effect.

## Data availability statement

The original contributions presented in this study are included in the article/supplementary material, further inquiries can be directed to the corresponding authors.

## Ethics statement

The studies involving human participants were reviewed and approved by Universiti Malaya Research Ethics Committee (Non-medical), University of Malaya. Written informed consent to participate in this study was provided by the participants’ legal guardian/next of kin.

## Author contributions

NW was responsible for writing, collect, and analysis the data. QW was responsible for design the work, helping revise the manuscript, providing edits for the revision, and proofreading. XL was revised the manuscript and provide substantial consultant on manuscript writing. MM was re-analysis of the data and did the English language checking. ZS was checked the language of the article. All authors contributed to the article and approved the submitted version.
